# Assessment of Antibiofilm Potencies of Nervonic and Oleic Acid against *Acinetobacter baumannii* Using In Vitro and Computational Approaches

**DOI:** 10.3390/biomedicines9091133

**Published:** 2021-09-01

**Authors:** Sagar Kiran Khadke, Jin-Hyung Lee, Yong-Guy Kim, Vinit Raj, Jintae Lee

**Affiliations:** School of Chemical Engineering, Yeungnam University, Gyeongsan 38541, Korea; 21850141@ynu.ac.kr (S.K.K.); jinhlee@ynu.ac.kr (J.-H.L.); yongguy7@ynu.ac.kr (Y.-G.K.); drvinitraj@ynu.ac.kr (V.R.)

**Keywords:** *Acinetobacter baumannii*, biofilm formation, AbaI, computational studies, fatty acid, nervonic acid, virulence, quorum sensing, antibiofilm agents

## Abstract

*Acinetobacter baumannii* is a nosocomial pathogen, and its biofilms are tolerant to desiccation, nutrient starvation, and antimicrobial treatment on biotic and abiotic surfaces, tissues, and medical devices. Biofilm formation by *A. baumannii* is triggered by a quorum sensing cascade, and we hypothesized that fatty acids might inhibit its biofilm formation by interfering with quorum sensing. Initially, we investigated the antibiofilm activities of 24 fatty acids against *A. baumannii* ATCC 17978 and two clinical isolates. Among these fatty acids, two unsaturated fatty acids, nervonic and oleic acid, at 20 μg/mL significantly inhibited *A. baumannii* biofilm formation without affecting its planktonic cell growth (MICs were >500 μg/mL) and markedly decreased the motility of *A. baumannii* but had no toxic effect on the nematode *Caenorhabditis elegans*. Interestingly, molecular dynamic simulations showed that both fatty acids bind to the quorum sensing acyl homoserine lactone synthase (AbaI), and decent conformational stabilities of interactions between the fatty acids and AbaI were exhibited. Our results demonstrate that nervonic and oleic acid inhibit biofilm formation by *A. baumannii* strains and may be used as lead molecules for the control of persistent *A. baumannii* infections.

## 1. Introduction

In response to different environmental stimuli, bacteria transition between planktonic and sessile states. Biofilms are matrix-enclosed communities and enable bacteria to resist host defenses and antibiotics [1,2], and thus bacteria in biofilms can endure for extended periods and are difficult to eradicate in hospital settings [1]. *Acinetobacter baumannii* was ranked by the World Health Organization as a highest priority critical pathogen in 2017 and the most successful multidrug-resistant ESKAPE organism (*Enterococcus faecium*, *Staphylococcus aureus*, *Klebsiella pneumoniae*, *A. baumannii*, *Pseudomonas aeruginosa*, *Enterobacter* organisms) [3,4,5]. Moreover, *A. baumannii* causes a wide range of infections, including pneumonia, bacteremia, endocarditis, osteomyelitis, meningitis, and severe nosocomial wound infections, urinary tract infections, bloodstream, skin, and other soft tissue infections [6,7,8]. Because *A. baumannii* possesses an array of acquired antibiotics resistance mechanisms, and as its natural habitat has not been well-defined, the treatment of its infections is challenging. Moreover, 5% to 10% of *A. baumannii* infections are hospital-acquired infections [4,6].

In *A. baumannii,* several factors, such as cell density sensing and protein glycosylation systems, nutrients, concentrations of free cations, poly-β-(1-6)-N-acetyl glucosamine extracellular polysaccharide, auto-inducer synthase, and biofilm-associated proteins, are involved in biofilm formation and maturation [1,9]. Furthermore, the expression of usher- chaperone assembly for pili production is responsible for motility and cell attachment and is required for successful biofilm development [10,11]. These virulence determinants help *A. baumannii* to adhere to eukaryotic cells and abiotic surfaces and promote invasion and eukaryotic cell death [12]. The *A. baumannii* quorum sensing (QS) system consists of *AbaIR* and AbaI (acyl homoserine lactone synthase) quorum sensing and its regulator AbaR, which are required for the production and regulation of 3-hydroxy-dodecanoyl-_L_-homoserine lactone [13,14]. The *AbaI* and *AbaR* genes are essential for biofilm formation and motility [15]. However, AbaR is viewed as an important target for *A. baumannii* biofilm inhibitors [16]. Recently, Tang et al. (2020) reported that the *AbaI* gene of *A. baumannii* plays a major role in the development of antibiotic resistance [14]. Additionally, targeting the AHL synthase enzyme AbaI could provide an effective strategy for attenuating virulence in *A. baumannii*, whereas the receptor *AbaR* may show unpredictable consequences [17]. As AbaI produces AHLs by incorporating S-adenosyl methionine (SAM) and acyl–acyl carrier proteins [18], the active binding pocket of AbaI with SAM (a natural ligand to AbaI) may open new avenues for the scientific community to search for a QS inhibitor against *A. baumannii.* Hence, these aforementioned observations suggest that AbaI can be used as a potential molecular target to discover new antibiofilm molecules against the biofilm of *A. baumannii*.

Fatty acids are widespread in animals, plants, and microbes, and more than 70 naturally occurring fatty acids have been identified to date [19,20]. Furthermore, studies suggest that fatty acids selectively inhibit or disrupt biofilm formation by various microorganisms, including *S. aureus*, *P. aeruginosa*, *Candida albicans*, *Serratia marcescens*, *Burkholderia cenocepacia*, and *Vibrio* spp. [21,22,23,24,25,26,27]. The antimicrobial and antibiofilm activities of fatty acids are largely dependent on the suppressions of quorum sensing genes related to virulence and other non-quorum sensing targets [20]. Although the underlying mechanisms responsible for the biofilm inhibitory effects by fatty acids have remained unclear, it is known that fatty acids act as antibiofilm agents at sub-MIC concentrations, whereas they affect multiple cellular targets and have nonspecific antimicrobial effects at higher concentrations [20]. Fatty acids also have beneficial effects against cancerous cells, neurodegenerative diseases, joint and bone diseases, coronary disease, and even depression [28,29,30]. Interestingly, fatty acids are structurally similar to a bacterial diffusible signaling factor [31], and unsaturated fatty acids such as myristoleic and palmitoleic acid have been reported to prevent the expression of *AbaR* in *A. baumannii* [32]. To date, no comprehensive study of *A. baumannii* AbaI and fatty acids is reported.

By observing the significant roles of AbaI presented above in the biofilm formation of *A. baumannii* and the role of fatty acids as QS inhibitors in several microorganisms, we hypothesized that these fatty acids may work as potent antibiofilm agents against *A. baumannii* by binding to the active pocket of AbaI, similar to SAM. To address this hypothesis, we selected 24 fatty acids for an in vitro screening against the biofilm formation of *A. baumannii.* Further, to confirm the protein binding of AbaI to the best in vitro target molecules, nervonic, oleic, and myristoleic acids were tested by in silico and complex stabilities of AbaI–fatty acids, supported by molecular dynamic simulation, to reveal the molecular mechanism of these fatty acids with AbaI protein. In addition, we performed in vivo cytotoxicity assays with nervonic, oleic, and myristoleic acids in *Caenorhabditis elegans*.

## 2. Materials and Methods

### 2.1. Ethics Statement

All experiments were carried out in accordance with relevant ethical guidelines and regulations. We further confirm that all experiments were either approved by the ethical committee of Yeungnam University or that there were no permission requirements.

### 2.2. Reagents, Bacterial Culture and Growth Rate Measurements

**Figure 1:**
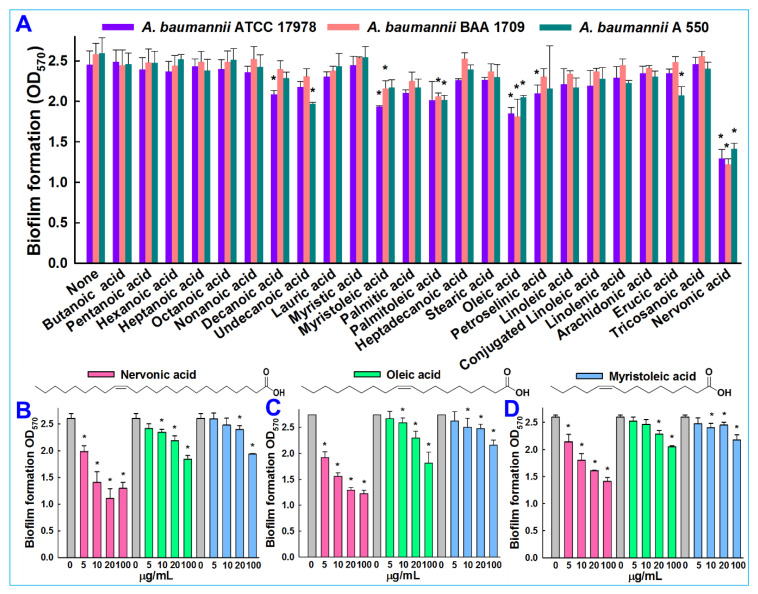
Impacts of various fatty acids on biofilm formation by the three *A. baumannii* strains in the presence of fatty acids at 100 μg/mL (**A**), The structures and antibiofilm activities of the selected fatty acids against the three strains of *A. baumannii* were quantified against *A. baumannii* ATCC 17978 (**B**), *A. baumannii* BAA 1709 (**C**), and *A. baumannii* A 550 (**D**). Error bars indicate standard deviations. *, *p* < 0.05 versus non-treated controls.

Chemicals including 24 fatty acids—butanoic acid (C4:0), pentanoic acid (C5:0), hexanoic acid (C6:0), heptanoic acid (C7:0), octanoic acid (C8:0), nonanoic acid (C9:0), decanoic acid (C10:0), undecanoic acid (C11:0), lauric acid (C12:0), myristic acid (C14:0), myristoleic acid (C14:1), palmitic acid (C16:0), cis-9-hexadecenoic acid (C16:1), heptadecanoic acid (C17:0), stearic acid (C18:0), oleic acid (C18:1), petroselinic acid (C18:1), linoleic acid (C18:2), conjugated linoleic acid (C18:2), linolenic acid (C18:3), arachidonic acid (C20:4), erucic acid (C22:1), tricosanoic acid (C23:0), and nervonic acid (C24:1) (Figure 1A)—and crystal violet were purchased from either Sigma-Aldrich (St. Louis, MO, USA), Cayman Chemicals (Ann Arbor, MI, USA), or TCI Co. (Tokyo, Japan). *A. baumannii* ATCC 17978 was acquired from American Type Culture Collection (ATCC), and two other clinical isolates of *A. baumannii* (ATCC BAA 1709 and A 550) [33], isolated from burn patients at the National Rehabilitation Institute of Mexico, were obtained as a gift from Prof. Garcia Contreras Rodolfo, UNAM, Mexico. For all experiments, *A. baumannii* strains were cultured under aerobic conditions at 37 °C in tryptic soy broth (TSB; Becton Dickinson, Sparks, MD, USA). Fatty acids were dissolved in dimethyl sulfoxide (DMSO). DMSO (0.1% (*v*/*v*)) was used as the negative control; at this concentration, it did not affect bacterial growth or biofilm formation.

Initially, bacterial strains were taken from −80 °C glycerol stock and streaked onto tryptic soy agar plates. Single fresh colonies were inoculated into TSB (2 mL) in 14 mL round bottom tubes and incubated at 37 °C and 250 rpm. For cell growth experiments, bacteria were reinoculated in 96-well plates containing TSB medium (1:100 dilution) and treated with or without nervonic, oleic, or myristoleic acid (positive control) at 20 µg/mL and incubated at 37 °C for 24 h. Growth was assessed by spectrophotometry at OD_620_. The minimum inhibitory concentrations (MICs) of fatty acids were determined as per CLSI guidelines [34]. Results quoted are the averages of at least two independent cultures.

### 2.3. In Vitro Screening of Fatty Acids against A. baumannii Biofilm

Overnight cultures of *A. baumannii* were inoculated at a dilution of 1:100. All 24 fatty acids were initially tested at 100 μg/mL for antibiofilm activity. Static biofilms were prepared in 96-well polystyrene plates (COSTAR, Corning Incorporated, Kennebunk, ME, USA), as previously reported [35]. Briefly, cells were inoculated into TSB (total volume 300 μL) and cultured with or without nervonic, oleic, or myristoleic acid at 0, 5, 10, or 20 μg/mL for 24 h without shaking at 37 °C. Biofilms formed were stained with 0.1% crystal violet and dissolved in 95% ethanol. Absorbances were measured at OD_570_ to quantify total biofilm formations. Results are the average absorbances of at least six replicate wells.

### 2.4. Measurements of Surface Motility

Tryptone yeast extract (TYE) agar plates were pierced to plate bottoms with pipettes containing 1 µL of overnight cultures. The agar plate contained 0.4% agarose, 1% tryptone, and 0.5% yeast extract. Motilities were measured in the presence or absence of nervonic, oleic, or myristoleic acid at concentrations of 10 and 20 µg/mL, respectively. Plates were incubated for 24 h at 37 °C, and the diameters of halos produced by cells traveling across agar surfaces were measured [35]. Measurements were taken from at least three independent cultures.

### 2.5. Microscopic Architecture of A. baumannii Biofilm

Scanning electron microscopy was used to examine biofilms on nylon membranes, as previously described [36]. Briefly, a nylon membrane was cut into 0.5 × 0.5 cm pieces and pieces were placed in 96-well plates containing *A. baumannii* grown in TSB medium with or without nervonic, oleic, or myristoleic acid (20 μg/mL) and incubated for 24 h at 37 °C. Cells that adhered to nylon membranes for 24 h were fixed with glutaraldehyde (2.5%) and formaldehyde (2%), postfixed using osmium tetroxide, and dehydrated using an ethanol series (50%, 70%, 80%, 90%, 95%, and 100%) and isoamyl acetate. After critical-point drying, cells were sputter-coated with palladium/gold and imaged using an S-4100 scanning electron microscope (Hitachi, Tokyo, Japan) at a voltage of 15 kV.

### 2.6. Assessment of the Cytotoxicity of Fatty Acids against C. elegans

*C. elegans* assays were performed as reported previously [19]. In brief, noninfected *C. elegans fer-15*; *fem-1* worms (n = ~ 20–30) were pipetted into single wells of a 96-well plate suspended in M9 buffer. Nervonic, oleic, or myristoleic acid (50 µg/mL) was then added to a final volume of 300 µL. Nematodes were incubated for 4 days at 25 °C, and viabilities were determined using an iRiS^TM^ Digital Cell Imaging System (Logos BioSystems, Anyang, Korea) by exposing worms to LED or UV LED light for 10–30 s.

### 2.7. Homological Modeling of Three-Dimensional Protein Structure

Homology modeling is used to build 3D structures of proteins to investigate protein–ligand interactions. The amino acid sequence of AbaI shown in the Supplementary Materials was adopted from the GenBank database under accession no. EU334497 [13] and was used for the homological modeling of AbaI using the Schrodinger [37] and Swiss [38] models, because the protein structure of AbaI was not available as a 3D structure in pdb format. Similarly, Smith et al. also reported the genome of *Acinetobacter baumannii* ATCC 17978, which showed 96% identity (GenBank accession number NC_009085.1) with the AbaI template of *Acinetobacter baumannii* M2 [13,39].

### 2.8. Three-Dimensional Structure Validation of Model Protein

To evaluate the quality of the model, it is essential to know whether built-up protein has a good-quality model. The validation of the structure of the constructed AbaI was analyzed using Ramachandran plots. The Ramachandran plot (Supplementary Figure S1B) showed that the constructed protein had 93.2% of residues in the more favorable region, 6% in allowed regions, and only 0.8% in disallowed regions, which indicated that the homological model of the constructed protein was of good quality.

### 2.9. Ligands and Acyl Homoserine Lactone Synthase Protein Collection

We used the protein preparation wizard tool in the Schrodinger suite to refine the protein structure of AbaI obtained by homological modeling, and essential hydrogens were added. In addition, the 2D structures of nervonic, oleic, and myristoleic acid were downloaded in simulation description format (SDF) from PubChem (https://pubchem.ncbi.nlm.nih.gov/, accessed on 14 October 2020).

### 2.10. Preparation of Ligands and Computational Screening

Ligand structures were prepared using LigPrep tools in the Schrodinger suite and optimized for minimum energy using the density functional theory (DFT) approach [40,41]. Conformations and bond orders were minimized and refined using the OPLS 2005 force field [42]. Prepared ligands were subjected to analysis for computational screening with the active binding pocket of AbaI. Initially, the binding active pocket of AbaI was predicted by the CASTp server [43]. Furthermore, the CASTp server-predicted active binding site of AbaI was made into a grid for the re-confirmation of the active binding pocket of protein by the VINA random method, with 25 runs between the ligand and AbaI. Later, the predicted active site was assigned for a final grid by molecular screening by treating drug molecules as rigid entities and the receptor as a flexible entity [44,45]. To ensure the reliability, validity, and reproducibility of docking results, molecular docking was performed using AUTODOCK [46] and VINA [47]. Binding energies and interactions between nervonic, oleic, or myristoleic acid and AbaI were determined using a computational approach, as previously described [46]. Further, a comparative molecular docking study was employed using SAM (natural ligand to AbaI) (positive control) and two non-active fatty acids, linolenic acid or tricosanoic acid, with the same assigned active site of AbaI protein to confirm the conformation binding of these molecules. BIOVIA Discovery Studio Visualizer was used to capture interactions between fatty acids and AbaI.

### 2.11. Estimation of ADME Property of Assigned Fatty Acids

The drug-likeness parameters of selected fatty acids were evaluated using the Schrodinger ADME prediction tool and Swiss ADME. According to the Lipinski rule, an orally active pharmaceutical agent should have a molecular weight ≤500 g/mol, a Log P of ≤5, ≤5 hydrogen bond-donating atoms, ≤10 hydrogen-bond-accepting atoms, and a topological polar surface of ≤140 [48]. Drug-likeness analysis suggested that nervonic, oleic, or myristoleic acid followed the considerable range of the Lipinski rule.

### 2.12. Molecular Dynamic (MD) Simulations and Energy Calculations

We performed an explicit solvent MD simulation in water with nervonic, oleic, or myristoleic acid complexes with AbaI protein using YASARA dynamic software, as previously reported [49,50]. Briefly, a periodic simulation cell boundary size of X = 61.30 Å, Y = 82.84 Å, and Z = 52.14 Å was assembled around the entire complex system and occupied with explicit water with a density of 0.997 g/L. Cl and Na ions were arbitrarily employed to attain charge neutrality. Pka values were employed for the side chains of Asp, His, Glu, and Lys residues [51]. The AMBER14 molecular dynamic force field was selected under physiological conditions of 298 K, 0.9% NaCl, and pH 7.4 [52]. Further, system energies were minimized initially by steepest descent minimization [53]. MD simulation runs of more than 100.3 ns for all targeted fatty acids were implemented at constant temperature and pressure, and all trajectories were saved each 250 ps for further analysis. These trajectories were examined by the YASARA template files, namely “md_analysis.mcr” and “md_analyzebindenergy.mcr” [49]. The root mean square deviations (RMSDs) and binding energies of complexes were calculated and represented graphically using Sigma Plot 10.0. Nervonic, oleic, and myristoleic acid with AbaI binding conformations were represented with Discovery Studio visualization software.

### 2.13. Statistical Analysis

Replication numbers for assays are provided above, and results were expressed as means ± standard deviations. The statistical analysis was performed by a one-way ANOVA followed by Dunnett’s test using SPSS version 23 (SPSS Inc., Chicago, IL, USA). *p* values of <0.05 were considered significant. Asterisks are used to denote significant differences between treated and untreated samples.

## 3. Results

### 3.1. In Vitro Validation of Antibiofilm Activity of Fatty Acids against A. baumannii

The antibiofilm potencies of the 24 fatty acids at 100 μg/mL (Figure 1A) were initially investigated on *A. baumannii* ATCC 17978 and two clinical isolates (BAA 1709 and A 550). In the initial screening, six fatty acids—decanoic, myristoleic, petroselinic, palmitoleic, oleic, and nervonic acid—displayed antibiofilm activities against *A. baumannii* ATCC 17978. Although slightly different responses were observed between strains, ATCC 17978 and BAA 1709 appeared to be more susceptible to the *cis* configuration middle and long-chain fatty acids (≤10 carbon atoms). Previously, it was reported that myristoleic and palmitoleic acid decreased *A. baumannii* ATCC 17978 biofilm formation [32], which was consistent with the results observed in this study. Notably, the long chain unsaturated nervonic acid inhibited 47%, 52%, and 45% of biofilm formation against *A. baumannii* ATCC 17978, BAA 1709 and A 550, respectively (Figure 1A).

**Figure 2:**
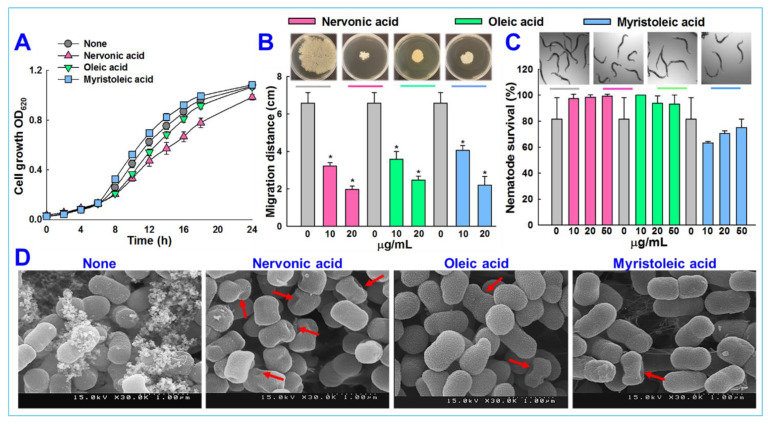
*A. baumannii* ATCC 17978 cell growth was investigated in the presence of nervonic, oleic, or myristoleic acid at 20 μg/mL (**A**), *A. baumannii* ATCC 17978 motility was assessed in the presence of fatty acid at 20 μg/mL after incubation for 24 h (**B**), the cytotoxicity of fatty acid was evaluated against *C. elegans* (**C**), *A. baumannii* ATCC 17978 biofilm formations on nylon membrane were observed by SEM in the presence of fatty acid at 20 μg/mL. Red arrows indicate dents in cells and scale bars represent 1 µm (**D**). Error bars indicate standard deviations. *, *p* < 0.05 versus non-treated controls.

Nervonic, oleic, and myristoleic acid dose-dependently inhibited biofilm formation for all three *A. baumannii* strains. For example, nervonic, oleic, and myristoleic acid (positive control) at 20 μg/mL inhibited biofilm formation by *A. baumannii* ATCC 17978 by up to 57%, 16%, and 7%, respectively (Figure 1B). Similar results were observed for the two clinical isolates (Figure 1C,D). Planktonic cell growths were measured to investigate the antimicrobial activities of the three fatty acids (Figure 2A). None of the three fatty acids inhibited the planktonic growth of *A. baumannii* at 20 μg/mL, and their minimum inhibitory concentrations (MICs) were all above 500 μg/mL. These results showed that three fatty acids only prevented biofilm formation by *A. baumannii* strains at sub-inhibitory concentrations, indicating nervonic and oleic acid may be less prone to the development of drug resistance in *A*. *baumannii*.

### 3.2. Nervonic Acid Impaired A. baumannii Motility

We also examined whether the three fatty acids inhibited other quorum sensing-regulated phenomena, such as motility. The surface motility of *A. baumannii* ATCC 17978 on 0.4% agarose was assessed by measuring mean halo diameters, and after 24 h, these were 1.9 ± 0.1, 2.4 ± 0.2, and 2.2 ± 0.4 cm for nervonic, oleic, and myristoleic acid, respectively, at a concentration of 20 μg/mL and 6.5 ± 0.5 cm for non-treated controls (Figure 2B). This is interesting since pili play a role both in biofilm formation and motility, which were inhibited by nervonic and oleic acid.

### 3.3. Microscopic Observations of Biofilm Inhibition by A. baumannii

The biofilm inhibitory activities of nervonic, oleic, and myristoleic acid (positive control) at 20 μg/mL against *A. baumannii* ATCC 17978 were also examined by SEM. Fully grown biofilms containing bacterial cells embedded in an exopolysaccharide (EPS) matrix on nylon membranes were observed in the absence of fatty acids (None) (Figure 2D). EPS formation decreased in the presence of nervonic or oleic acid to a greater extent than in the presence of myristoleic acid, and the observed biofilm inhibition was in line with the results of the crystal violet biofilm formation assay (Figure 1B). Interestingly, nervonic acid treatment caused dents on cell surfaces (Figure 2D), while oleic and myristoleic acid slightly reduced cell aggregation with few dents.

### 3.4. Cytotoxicity of Fatty Acids in the Nematode Caenorhabditis elegans

We conducted an *in vivo* study to investigate the cytotoxic activities of nervonic, oleic, and myristoleic acid using a *C. elegans* model. This nematode model is widely used to identify the toxicity of chemicals. After a trial for 4 days, nervonic, oleic, and myristoleic acid-treated nematodes showed no chemical toxicity and displayed similar trends to the non-treated controls (Figure 2C), confirming that nervonic and oleic acid were non-toxic to worms.

### 3.5. Molecular Docking of Fatty Acids with AbaI and Estimation of Molecular Interactions

**Figure 3:**
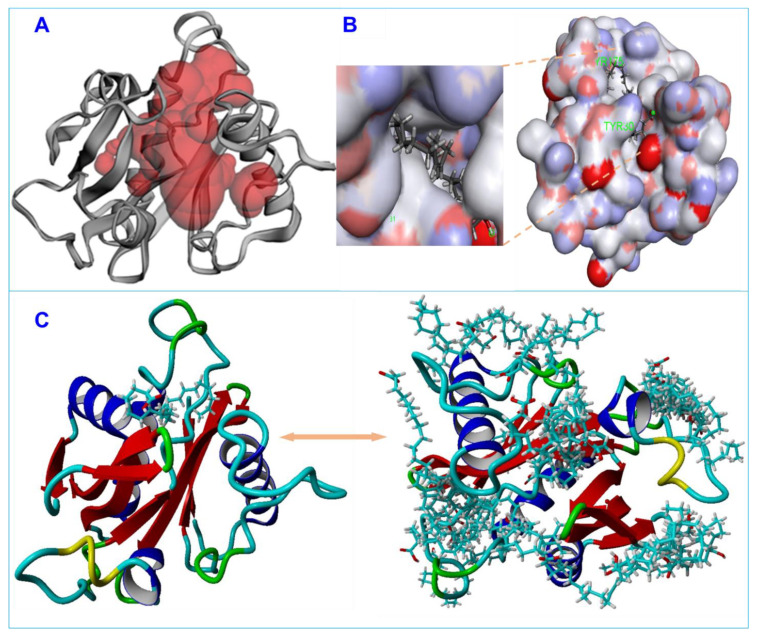
The putative predicted active pocket of ligand binding on the rigid surface of AbaI is shown in red (**A**), stable conformation of the ligand–receptor complex (**B**), and 25 molecular docking runs of nervonic acid with AbaI receptor (**C**).

**Figure 4:**
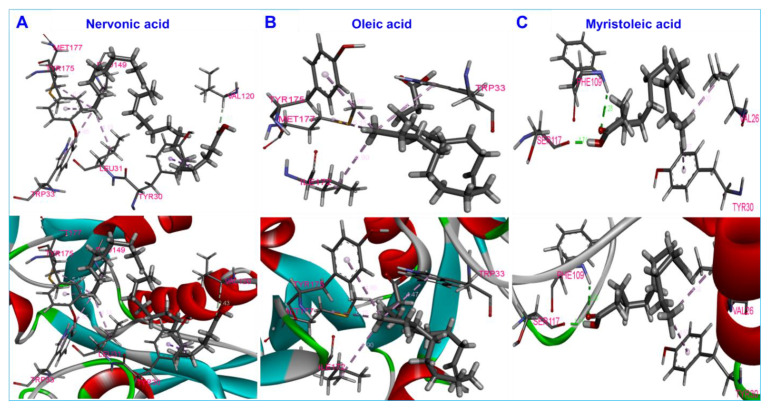
Interactions of ligands with AbaI receptor protein with nervonic (**A**), oleic (**B**), and myristoleic acid (**C**).

Molecular docking was carried out to investigate the interactions of nervonic, oleic, and myristoleic acid with homologized AbaI protein (Supplementary Figure S1A). Additionally, absorption, distribution, metabolism, and excretion (ADME) analysis was performed to analyze the pharmacokinetic profile of nervonic, oleic, and myristoleic acid, with pharmacokinetic parameters shown to be in a decent range as described by the Lipinski rule [48] (Supplementary Table S1, Supplementary Figure S1C–E). The binding domain of the active pocket of AbaI was confirmed using the CASTp server (Figure 3A,B), and random 25 docking with AbaI was carried out to re-confirm the accuracy of the active pocket predicted by the CASTp server (Figure 3C). The binding affinities of nervonic, oleic, and myristoleic acid with the predicted active binding site of AbaI fell in the range −5.0 to −5.9 kcal/mol (AUTODOCK). Redocking studies using VINA produced similar binding energies (Table 1). Nervonic acid exhibited a binding energy of −5.3 kcal/mol, with the active binding domain of AbaI being similar to the previously reported antibiofilm myristoleic acid, with −5.9 kcal/mol binding energy [32]. Furthermore, SAM as a positive control was used and showed a conformational binding energy of about −6.7 kcal/mol, while two non-active fatty acids, linolenic or tricosanoic acid, exhibited inferior binding conformation with AbaI (−4.7 and −4.8 kcal/mol respectively) (Table 1) (Supplementary Figure S2). Nervonic acid formed seven π–π bonds with amino acid residues of AbaI and formed a conformation binding like SAM, interacting with Leu31, Pro149, Tyr175, and Met177 of AbaI (Figure 4A). In contrast, oleic acid had a binding energy of −5.07 kcal/mol, formed four π–π bonds with AbaI, and also interacted with Ile172, Tyr175, and Met177, similar to nervonic acid and SAM (Figure 4B). However, myristoleic acid formed two π–π bonds and two hydrogen bonds with Val26, Tyr30, Phe109, and Ser117 amino acid residues of AbaI (Figure 4C). Among these four residues, none of them were similar to SAM docking. Overall, the comparative results of the molecular docking of nervonic acid and oleic acid were found to be more coherent than for non-active linolenic or tricosanoic acid. Additionally, the common amino acid binding shows that nervonic acid and oleic acid bind with similar binding pockets to SAM.

### 3.6. Conformational Stability of Fatty Acids with AbaI by Molecular Dynamic (MD) Simulation

**Figure 5:**
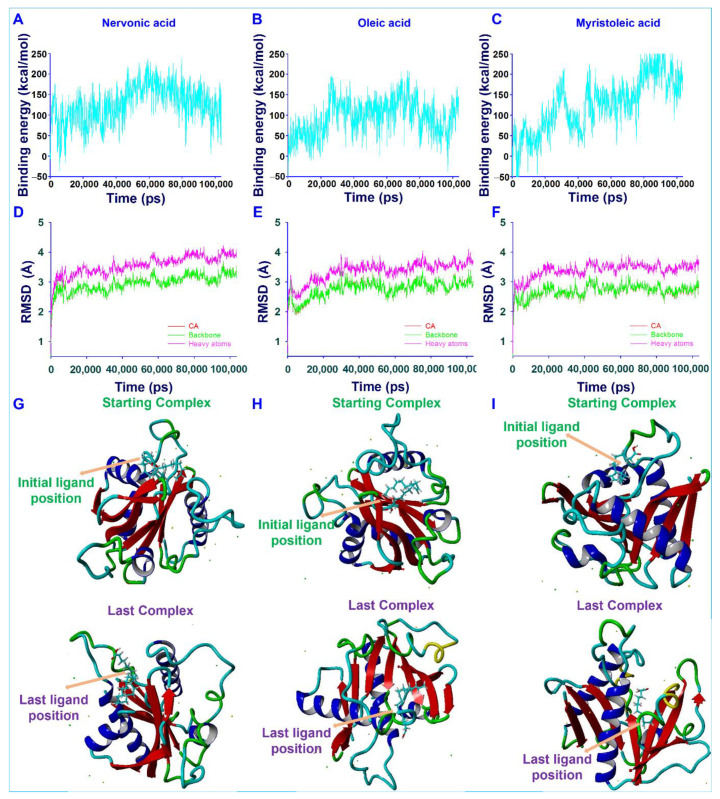
Ligand–AbaI complex stability studies to reveal the interactions. Binding energies (**A**–**C**) and RMSD of nervonic, oleic, and myristoleic acid, respectively, as determined by MD simulation of YASARA (**D**–**F**). Ligand–AbaI complex comparative binding positions of nervonic (**G**), oleic (**H**), and myristoleic acid (**I**) with AbaI as displayed before and after 100.3 ns MD simulations.

The motions and conformation stabilities of AbaI with nervonic, oleic, and myristoleic acid complexes were evaluated under solution conditions by MD simulation. Complexes with high binding energy were estimated to reveal the amino acid interactions of ligands over time. Generated trajectories were analyzed to determine ligand binding energies and root-mean-square deviations (RMSDs) of ligand–receptor complexes over a time of 100.3 ns. The average binding energies of nervonic (Figure 5A), oleic (Figure 5B), and myristoleic acid (Figure 5C) were found to be 126.52, 95.93, and 125.24 kcal/mol, respectively. These positive binding energies suggested that nervonic acid binds strongly compared to oleic and myristoleic acid with AbaI protein throughout MD runs. The RMSD of the nervonic acid–AbaI complex (Figure 5D) exhibited equilibrium trajectories after 2000 ps, while oleic (Figure 5E) and myristoleic acid (Figure 5F) showed equilibrium trajectories after 3000 and 2100 ps, respectively. However, the nervonic acid–AbaI complex showed better complex conformational stability during 100.3 ns MD runs than oleic or myristoleic acid. In addition, the initial position of nervonic acid (Figure 5G) in its complex with AbaI was more similar to its final position after 100.3 ns MD simulation than oleic (Figure 5H) or myristoleic acid (Figure 5I).

### 3.7. Plausible Mechanism for the Inhibition of A. baumannii Quorum Sensing by Nervonic Acid

The *A. baumannii* QS system requires AbaI and AbaR, which are homologous to the LuxIR system present in other Gram-negative bacteria. AbaI is encoded by the *AbaI* gene and functions as an autoinducer synthase to produce AHL compounds. Autoinducer synthase enzymes utilize SAM, which binds to acyl–acyl carrier protein (ACP) by forming an amide bond between the amino group of homocysteine and the acyl group of ACP to produce AHL compounds, subsequently resulting in the release of methylthioadenosine [54,55]. Lux-box (CTGTAAATTCTTACAG) is located 67 bp upstream of the *AbaI* gene start codon and is the binding site responsible for the production of structurally different AHL compounds in a positive feedback loop manner by AbaI and acyl transferases [13,14,56]. *AbaR* functions as receptor protein for the AHL compound, and the binding induces a cascade of reactions [18]. The *AbaI* gene product AbaI is essential for *A. baumannii* biofilm formation and motility [13,14,17].

**Figure 6:**
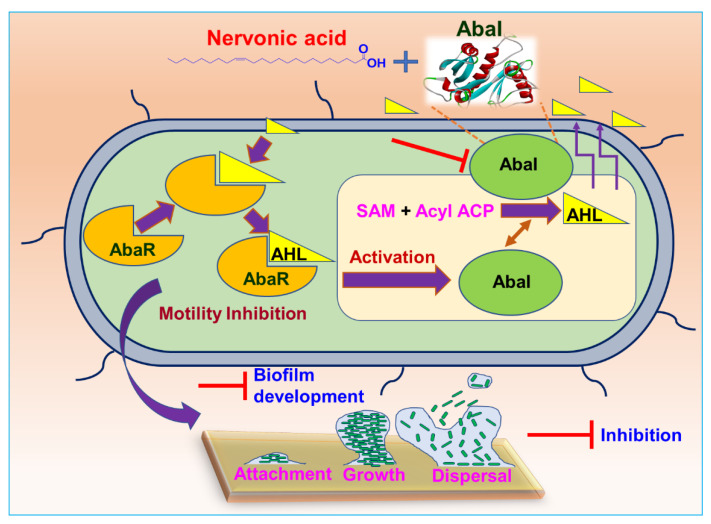
Plausible mechanism for the inhibition of *A. baumannii* quorum sensing by nervonic acid.

Structural similarities between nervonic, oleic, and myristoleic acid, the potent ability of myristoleic acid to interfere with the QS system [14,31,32], and our computational study and in vitro antibiofilm activity results for nervonic and oleic acid collectively suggest that these fatty acids might block AbaI protein (Figure 6) by binding with Leu31, Pro149, Ile172, Tyr175, and Met177 of AbaI, leading to the eradication of biofilm formation by *A. baumannii*.

## 4. Discussion

Fatty acids are ubiquitous amphiphilic molecules with a long carbon chain and terminal carboxyl group and are considered prospective contenders for use against recalcitrant pathogens [20]. Here, we report the biofilm inhibitory capacities of a series of fatty acids against the clinically relevant biofilm-forming bacterial pathogen *A. baumannii*. Among the tested 24 fatty acids, nervonic and oleic acid most efficiently reduced *in vitro* biofilm formation by three *A. baumannii* strains (Figure 1A–D). Nicole et al. (2018) reported that myristoleic acid at 20 μg/mL inhibited *A. baumannii* biofilm formation by 28% [32], while we found that myristoleic acid inhibited biofilm formation by 7% at the same concentration. Furthermore, myristoleic and palmitoleic acid impeded the motility of *A. baumannii* [32], which is interesting as pili play a role in biofilm formation and motility. In our study, motility was significantly reduced by nervonic acid as compared to oleic or myristoleic acid (Figure 2B). *A. baumannii* biofilm formation and motility depend on the synthesis of pili and are associated with type IV pili [10,11], which are structures assembled by CsuA/BABCDE, the pilus usher–chaperone secretion system under the control of A1S_2811 [57], and the *AbaI*-dependent quorum sensing pathway [57,58,59]. Thus, these comparative observations of the biofilm and motility inhibition of *A. baumannii* suggested that nervonic and oleic are leading molecules for further investigation. In addition, to reveal the plausible molecular mechanism of these fatty acids with AbaI protein, molecular docking was carried out, and we found that these fatty acids bind with AbaI via common amino acids Leu31, Pro149, Ile172, Tyr175, and Met177, indicating that these essential amino acids are the key residues for binding with the active pocket domain of AbaI. As a previous report suggests that SAM and acyl–acyl carrier protein substrates are essential for the synthesis of the auto-inducers (AHLs) [18], inspired by the significant role of SAM, we proposed that similar binding ligands to SAM with AbaI might work as a barrier for the synthesis of AHLs, leading to the inhibition of biofilm formation and virulence. In this regard, we searched for ligands which have the same binding conformation with AbaI, and the outcomes of the conformational binding of SAM with AbaI and their amino acid residues were similar to those of our tested binding of SAM with AbaI (Table 1), confirming the same conformational binding of nervonic and oleic acid to AbaI [60]. Among the bound amino acid residue of nervonic acid, four amino acid residues (Leu31, Pro149, Tyr175, and Met177) matched with those of SAM, and three amino acid residues (Ile172, Tyr175, and Met177) of oleic acid docking matched with SAM. Thus, similar to SAM, the binding conformation of nervonic and oleic acid with AbaI suggest that these fatty acids are possible lead molecules to design a potent AbaI inhibitor against *A. baumannii.* Further, the stabilities of complexes of these fatty acids with AbaI in MD simulation supported our hypothesis that nervonic acid specifically has better conformational stability and strongly binds with AbaI protein compared to oleic or myristoleic acid, along with presenting a similar initial and final position throughout MD runs (Figure 5). Hence, it can be suggested that the nervonic or oleic acid–AbaI complex might hamper the production of AHL molecules, disrupt the quorum sensing cascade, and render *A. baumannii* in the planktonic cell stage.

## 5. Conclusions

At present, novel antivirulence agents are required to address the challenges posed by drug-resistant microorganisms. In this regard, we addressed the screening of fatty acids against the biofilm of *A. baumannii* using in silico and in vitro approaches, which collectively suggested that nervonic and oleic acid act as suppressors of AbaI protein in *A. baumannii* and of biofilm formation without killing bacteria or *C. elegans*. The remarkable conformational stability of nervonic and oleic acid with AbaI protein helped us to understand the molecular mechanism of the biofilm inhibition of *A. baumannii* via AbaI deactivation. Additionally, the in vivo safety profiles and in vitro antibiofilm potencies of nervonic and oleic acid suggested that these molecules might be considered to treat persistent infections either singly or in combination as adjunctive treatments. This binding of fatty acids to AHL synthase through molecular simulation studies will help researchers to design or find new biomolecules targeting the QS network.

## Figures and Tables

**Table 1:** Binding energies of targeted ligands with AbaI. Amino acid residues essentially required for binding are colored blue.

Compounds	Receptor	Binding Energy (Kcal/mol)	Binding Energy (Kcal/mol)	Indicating Amino Acids	Bonds
		VINA	AUTODOCK		
Nervonic acid	AbaI	−5.3	−5.3	Tyr30, Leu31, Trp33, Val120, Pro149, Tyr175, Met177	7π–π
Oleic acid	AbaI	−5.07	−5.07	Trp33, Ile172, Tyr175, Met177	4π–π
Myristoleic acid	AbaI	−5.9	−5.9	Val26, Tyr30, Phe109, Ser117	2π–π, 2H
S-adenosyl methionine	AbaI	−5.9	−6.7	Leu31, Ile65, Ser105, Val107, Ser121, Ile128, Pro149, Leu150, Met170, Met171, Ile172, Gly174, Tyr175, Ser176, Met177	2π–π, 13H
Linolenic acid	AbaI	−4.9	−4.7	Leu31, Ala106, Val107, Pro149	2π–π, 2H
Tricosanoic acid	AbaI	−4.6	−4.8	Tyr30	2π–π

## Data Availability

Not applicable.

## Supplementary Materials

The following are available online at https://www.mdpi.com/article/10.3390/biomedicines9091133/s1, Amino acids sequence, Supplementary Figure S1: Homological modeling of acyl-homoserine-lactone synthase, Supplementary Table S1: Various physicochemical parameters of fatty acids to reveal the possible ADME properties, Supplementary Figure S2: Ligands with AbaI receptor protein interactions of S-adenosyl methionine (A), linolenic (B), and tricosanoic acid (C), References.
