# Supplementary material for: Assessment of Antibiofilm Potencies of Nervonic and Oleic Acid against Acinetobacter baumannii Using In Vitro and Computational Approaches
Source: Biomedicines. 2021 Sep 1;9(9):1133. doi: 10.3390/biomedicines9091133 (PMC8466663; doi:10.3390/biomedicines9091133)
Supplement: Supplementary file 1 [file biomedicines-09-01133-s001.zip › biomedicines-1325376-supplementary.pdf]

Supplementary Data

Assessment of antibiofilm potencies of nervonic and oleic acid against *Acinetobacter baumannii* using *in vitro* and computational approaches

Sagar Kiran Khadke[1], Jin-Hyung Lee[1], Yong-Guy Kim[1], Vinit Raj[1], Jintae Lee[1]*

[1]*School of Chemical Engineering, Yeungnam University, Gyeongsan, 38541, Republic of Korea*

***Corresponding author:** E-mail: jtlee@ynu.ac.kr

Phone: 82-53-810-2533, Fax: 82-53-810-4631

Amino acids sequence

Acyl-homoserine-lactone synthase > B0FLN1 (*A. baumannii*) 184 amino acids[1]

MNIIAGFQNNFSEGLYTKFKSYRYRVFVEYLGWELNCPNNEETRIQFDKVDTAYVV
AQDRESNIIGCARLLPTTQPYLLGEIFPQLLNGMPIPCSPEIWELSRFSAVDFSKPPSSSS
QAVSSPISIAILQEAINFAREQGAKQLITTSPLGVERLLRAAGFRAHRAGPPMMIDGYS
MFACLIDV

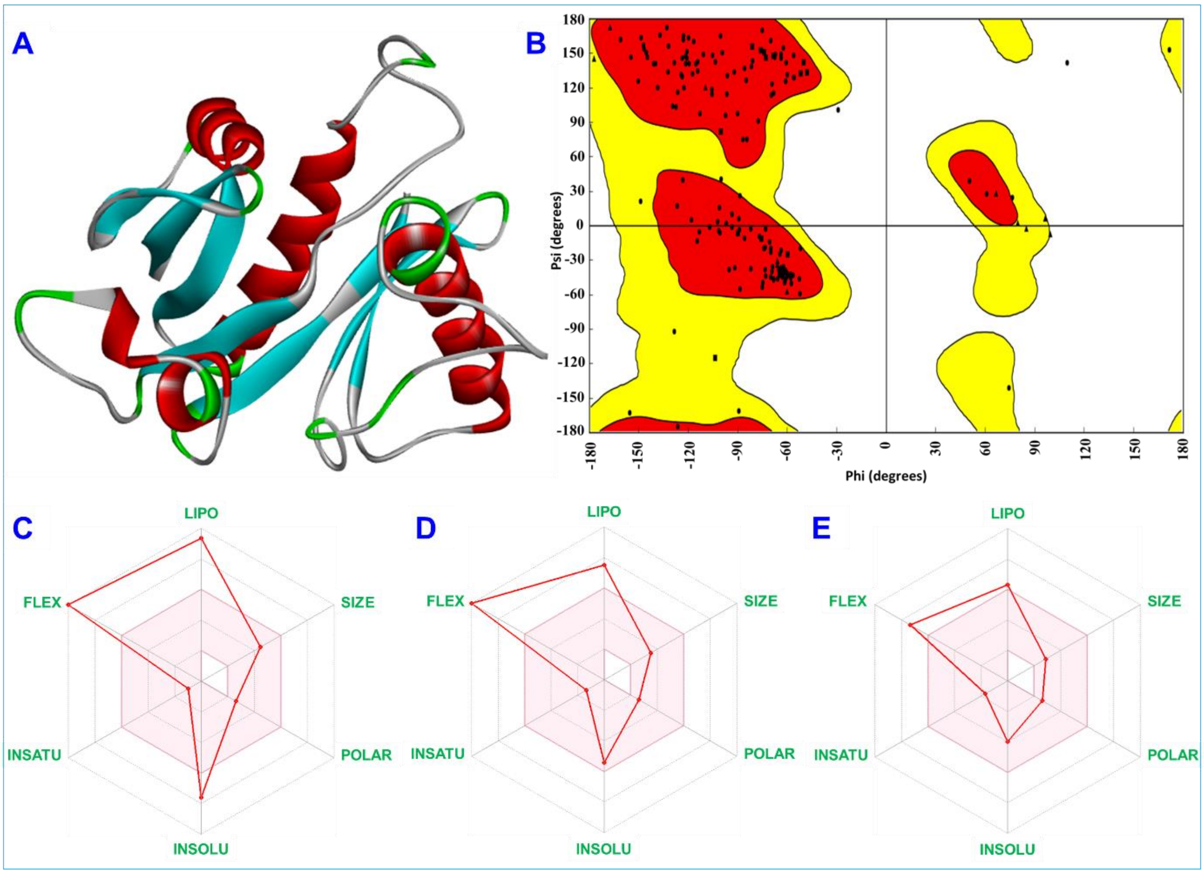

Supplementary Figure S1. Homological modeling of acyl-homoserine-lactone synthase: three-dimensional structure of acyl-homoserine-lactone synthase receptor **(A)**, conformational validation of acyl-homoserine-lactone synthase by Ramachandran plot **(B)**, ADME analysis for the selected long chain fatty acids using Swiss ADME server by using drug 2D structural formula for calculating the properties Lipophilicity (LIPO), size, polarity (POLAR), insolubility in water (INSOLU), instauration (INSATU), and flexibility (FLEX) nervonic acid (**C**), oleic acid (**D**), and myristoleic acid (**E**).

Supplementary Table S1. Various physicochemical parameters of fatty acids to reveal the possible ADME properties.

Parameters	Value		
	Nervonic acid	Oleic acid	Myristoleic acid
Formula	$C_{24}H_{46}O_2$	$C_{18}H_{34}O_2$	$C_{14}H_{26}O_2$
Molecular Weight	366.62	282.46	226.36
H-bond acceptors	2	2	2
H-bond donors	1	1	1
Topological Polar Surface Area	37.3	37.3	37.3
Gastrointestinal absorption	Low	High	High
Blood Brain Barrier permeability	No	No	Yes
Lipinski violations	1	1	0
Bioavailability Score	0.85	0.85	0.85
CYP2C19 inhibitor	No	No	No

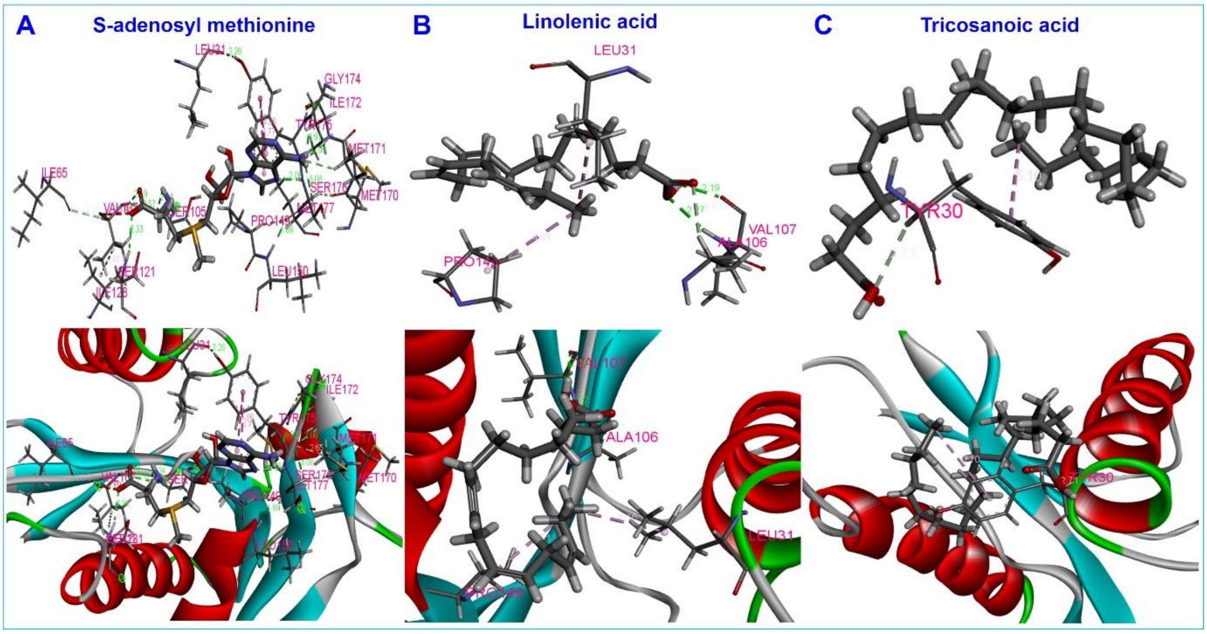

Supplementary Figure S2. Ligands with AbaI receptor protein interactions of s-adenosyl methionine **(A)**, linolenic **(B)**, and tricosanoic acid **(C)**.

References

1. Niu, C.; Clemmer, K.M.; Bonomo, R.A.; Rather, P.N. Isolation and characterization of an autoinducer synthase from *Acinetobacter baumannii*. *J. Bacteriol.* **2008**, *190*, 3386-3392, doi:10.1128/JB.01929-07.
